# Complete chloroplast genome sequence of *Taxillus chinensis* (Loranthaceae): a hemiparasitic shrub in South China

**DOI:** 10.1080/23802359.2019.1666680

**Published:** 2019-09-19

**Authors:** Bingbing Liu, Ying Zhang, Yancai Shi

**Affiliations:** a Institute of Loess Plateau, Shanxi University, Taiyuan, China;; b Guangxi Key Laboratory of Plant Conservation and Restoration Ecology in Karst Terrain, Guangxi Institute of Botany, Guangxi Zhuang Autonomous Region and Chinese Academy of Sciences, Guilin, China

**Keywords:** *Taxillus chinensis*, chloroplast genome, phylogenetic analysis

## Abstract

*Taxillus chinensis* (DC) Danser, which is a hemiparasitic shrub distributed in southern China and Southeast Asian countries, is wildly used in various traditional Chinese medicine prescriptions. In this study, the complete chloroplast genome of the *T. chinensis* was assembled from the whole genome Illumina sequencing data. The complete plastome is a typical quadripartite structure with a length of 121,305 bp, which contained two inverted repeats (IRs) of 22,460 bp separated by a large single-copy (LSC) and a small single copy (SSC) of 70,295 bp and 6090 bp, respectively. The plastome contains 106 genes, comprising 66 protein-coding genes, 28 tRNA genes, eight rRNA genes, and four processed pseudogenes. The overall GC content of the plastome is 37.4%, which is unevenly distributed across the whole chloroplast genome. The phylogenetic analysis shows that *T. chinensis* was closely related to the congeneric species *T. sutchuenensis.*

*Taxillus chinensis* (DC) Danser is a hemiparasitic shrub distributed in southern China (such as Guangxi, Guangdong, Fujian) and Southeast Asian countries (such as Vietnam, Thailand, Philippines) (Liu et al. 2019). Recorded hosts for this species include *Glyptostrobus pensilis*, *Hevea brasiliensis*, *Dimocarpus longan*, Moraceae spp. *Taxillus chinensis* is wildly used in various traditional Chinese medicine prescriptions such as the treatment of rheumatism, threatened abortion, hypertension, angina pectoris, stroke, and arrhythmia for many years in China (Li et al. 2017). To facilitate its genetic research and contribute to its utilization, here, we report and characterize the complete plastome of *T. chinensis* (GenBank accession number: MN080717, this study) based on Illumina paired-end sequencing data. Phylogenetic analysis was conducted, which will be useful for further studies on its chloroplast genetic engineering.

In this paper, *T. chinensis* was sampled from in Guangxi zhuang autonomous region of China, located at 110°03′49.46″E, 25°43′55.74 N. A voucher specimen (Y.-C. Shi et al. T108) was deposited in the Guangxi Key Laboratory of Plant Conservation and Restoration Ecology in Karst Terrain, Guangxi Institute of Botany, Guangxi Zhuang Autonomous Region and Chinese Academy of Sciences, Guilin, China. The experiment procedure is as reported in Zhang et al. (2019). Around 2 Gb clean data were used for the cp genome de novo assembly by the program NOVOPlasty (Dierckxsens et al. 2017) and direct-viewing in Geneious R11 (Biomatters Ltd., Auckland, New Zealand). Annotation was performed with the program Plann (Huang and Cronk 2015) and Sequin (http://www.ncbi.nlm.nih.gov/).

The plastome of *T. chinensis* was found to possess a total length 121,305 bp with the typical quadripartite structure of angiosperms, containing two inverted repeats (IRs) of 22,460 bp separated by a large single-copy (LSC) and a small single copy (SSC) of 70,295 bp and 6,090 bp, respectively. The cpDNA contains 106 genes, comprising 66 protein-coding genes, 28 tRNA genes, eight rRNA genes, and four processed pseudogenes. Among the annotated genes, six of them contain one intron (*atp*F, *rpo*C1, *clp*P*, pet*B*, pet*D, and *rpl*2), and two genes (*ycf*3 and *rps*12) contain two introns. The overall GC content of the plastome is 37.4%, which is unevenly distributed across the whole chloroplast genome.

To identify the phylogenetic position of *T. chinensis*, phylogenetic analysis was conducted. A neighbor-joining (NJ) tree with 1000 bootstrap replicates was inferred using MEGA version 7 (Kumar et al. 2016) from alignments created using the MAFFT (Katoh and Standley 2013) using plastid genomes of 16 species. It showed the position of *T. chinensis* was closely related to the species of *T. sutchuenensis* (Figure 1). Our findings will provide a foundation for facilitating its genetic research and contributing to its utilization in *Taxillus*.

**Figure 1.**
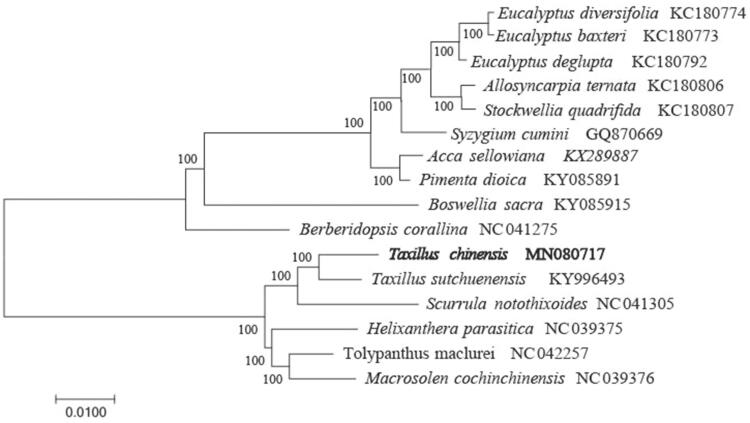
The neighbour-joining (NJ) tree based on the 16 chloroplast genomes. The bootstrap value based on 1000 replicates is shown on each node.
